# Impact of High Concentration Solutions on Hydraulic Properties of Geosynthetic Clay Liner Materials

**DOI:** 10.3390/ma5112326

**Published:** 2012-11-14

**Authors:** Qiang Xue, Qian Zhang, Lei Liu

**Affiliations:** State Key Laboratory of Geomechanics and Geotechnical Engineering, Institute of Rock and Soil Mechanics, Chinese Academy of Sciences, Wuhan 430071, China; E-Mails: zq_cersm@163.com (Q.Z.); lliu@whrsm.ac.cn (L.L.)

**Keywords:** geosynthetic clay liner materials, high concentration chemical solutions, chemical attack effect, hydraulic conductivity, hydration swelling

## Abstract

This study focuses on the impact of landfill high concentration solutions erosion on geosynthetic clay liner (GCL) materials permeability. The permeation tests on the GCL, submerged using different kinds of solutions with different concentrations, were carried out systematically by taking these chemical solutions as permeant liquids. Based on seasonal variations of ion concentrations in Chenjiachong landfill leachate (Wuhan Province), CaCl_2_, MgCl_2_, NaCl, and KCl were selected as chemical attack solutions to carry out experimental investigations under three concentrations (50 mM, 100 mM, 200 mM) and soak times (5, 10, and 20 days). The variation law of the GCL hydraulic conductivity under different operating conditions was analyzed. The relationship between GCL hydraulic conductivity, chemical solutions categories, concentrations, and soak times were further discussed. The GCL hydraulic conductivity, when soaked and permeated with high concentration chemical solutions, increases several times or exceeds two orders of magnitude, as compared with the permeation test under normal conditions that used water as the permeant liquid. This reveals that GCL is very susceptible to chemical attack. For four chemical solutions, the chemical attack effect on GCL hydraulic conductivity is CaCl_2_ > MgCl_2_ > KCl > NaCl. The impact of soak times on GCL hydraulic conductivity is the cooperative contribution of the liner chemical attack reaction and hydration swelling. A longer soak time results in a more advantageous hydration swelling effect. The chemical attack reaction restrains the hydration swelling of the GCL. Moreover, the GCL hydraulic conductivity exponentially decreases with the increased amplitude of thickness.

## 1. Introduction

Geosynthetic clay liner (GCL), as part of a landfill liner system, possesses good impermeability that enables it to isolate wastes from the surrounding environment. It also prevents, delays, and controls the surrounding soil and underground water from polluting caused by the migration of landfill leachate [1]. The Chinese Technical Specification of Municipal Sanitary Landfill indicates that the hydraulic conductivity of a landfill liner system must be lower than 1 × 10^−7^ cm/s to guarantee normal operations. However, landfill leachate is a highly concentrated chemical solution with complicated components that contains various ions such as Na^+^, K^+^, Ca^2+^ and Mg^2+^. Generally, the ions concentrations are as follows: Na^+^ and K^+^, 1000–2500 mg/L; Ca^2+^, as high as 2500 mg/L; Mg^2+^, as high as 2000 mg/L. In some circumstances, Ca^2+^ and Mg^2+^ almost achieved saturation [2]. For landfills in South China under a long-term chemical attack environment of highly concentrated leachate ions, the internal pores of the liner produce structural evolutions to enlarge pores. The changes in pores structure shorten the time leachate ions take to pass through or breakdown the liner. These processes decrease the service life of the liner and remove its partial or whole protection functions, thus polluting the surrounding water and soil environment. As a result, serious environmental and geological disasters are caused by the leakage of landfill leachate [3,4]. Therefore, carrying out experimental research on the permeability of GCL under chemical attack by high concentration solutions is of great practical significance to avoid the ineffective impermeability of a landfill liner system.

Recently, many scholars have carried out a series of researches on problems related to GCL application and obtained findings about the permeability [5,6,7,8,9,10,11,12,13,14,15,16]. Given that Chinese scholars have studied the field later and research on the permeability are mainly focused on traditional geotechnical engineering, there exists little research on the permeability of landfill liners [17,18]. Most current studies are concentrated on the permeability of GCL for water conservation or transportation, or carried out in environmental engineering on GCL permeability under chemical attack by relatively low concentration (≤20 mM) chemical solutions. And the experiments on the permeability are conducted using water as the permeant liquid after chemical attack for relative researches. Considering the strong chemical attack caused by ions of high concentration leachate, these experimental results are inconsistent with practical operating conditions. Therefore, combined with seasonal variation of ion concentrations in the typical leachate of the Chenjiachong landfill (Wuhan Province), high concentration (≥50 mM) chemical solutions containing Ca^2+^, Mg^2+^, Na^+^ and K^+^ were applied directly as permeant liquids in this paper to systematically study the impact of different chemical attack factors on GCL permeability and further discuss the variation law (changes in hydraulic conductivity) of GCL hydraulic conductivity with solution categories, concentrations, and soak times. This study aims to provide critical technical support and optimization to system parameter designs of landfill impermeability as well as applications concerning GCL.

## 2. Materials and Methods

### 2.1. Experimental Materials

The GCL applied in the experiment was a sodium bentonite liner composed of two layers of geotextile and one layer of sodium bentonite in the middle. The above and bottom layers of geotextile were textile fabric and nonwoven fabric, respectively, and were connected with the bentonite through a 6.48 mm-thick needle-punched fiber. The basic physical property of bentonite in the GCL is shown in Table 1.

**Table 1:** Properties of Na-bentonite used in the test.

Parameters	Na-bentonite
Specific gravity	2.76 ^a^
Montmorillonite content	76.3% ^b^
Mass per unit area	4.91 kg/m^2^
Void ratio	4.2
Dry density	0.62 g/cm^3^

Notes:^ a^ Using the pycnometer and a magnetic stirring device; ^b^ Based on x-ray diffraction (XRD) analyses.

In view of the ions components and concentrations in the leachate of Chenjiachong landfill (Wuhan Province), the typical ions Ca^2+^, Mg^2+^, Na^+^ and K^+^ were selected by preparing CaCl_2_, MgCl_2_, NaCl and KCl solutions with concentrations of 50, 100 and 200 mM, respectively. These chemicals solutions were used to study the variation law of the GCL hydraulic conductivity.

### 2.2. Experimental Method

The round GCL samples with a diameter of 300 mm were soaked in the prepared solutions for 5, 10 and 20 days respectively. The PN3230M environmental soil flexible wall permeameter, made by American GEOEQUIP, was used to test the solution permeability of the samples. Before each test, a pump was used to make vacuum for saturation of the specimen. The solutions applied during saturation and permeation were the same as that for soaking processes.

The confining pressure, up and down back pressures, and other factors the sample suffered were kept the same. The value was 550 kPa for the confining pressure, as well as 515 and 530 kPa for the up and down back pressures, respectively. In other words, the percolation pressure was 15 kPa and the average hydraulic gradient was set to 200. The room temperature was strictly controlled at 22 °C during the experiment to prevent the temperature factor from affecting the test results. The hydraulic conductivity mainly reflected the impact of solution categories, concentrations, and soak times on GCL permeability. Thirty-six samples were tested under different environmental conditions. The experimental conditions are shown in Table 2.

**Table 2:** Working condition of GCL specimens.

**Working condition**		**Soak for 5 days**			**Soak for 10 days**			**Soak for 20 days**		
		50 mM	100 mM	200 mM	50 mM	100 mM	200 mM	50 mM	100 mM	200 mM
solutions	CaCl_2_	50-5	100-5	200-5	50-10	100-10	200-10	50-20	100-20	200-20
	MgCl_2_	50-5	100-5	200-5	50-10	100-10	200-10	50-20	100-20	200-20
	NaCl	50-5	100-5	200-5	50-10	100-10	200-10	50-20	100-20	200-20
	KCl	50-5	100-5	200-5	50-10	100-10	200-10	50-20	100-20	200-20

The solution categories, concentrations, and soaking times were the three control factors of this experiment. Variations of the water volume in the inlet and outlet glass pipes on the pressure control panel were regularly read during the experiment. The hydraulic conductivity was regarded as stable when volumetric flow ratios of outlet relative inlet (Q_out_/Q_in_) was within 1.00 ± 0.25. Regular measurements of the pH and EC (electrical conductivity) of solutions in the outlet, compared with those in the inlet, were required. The samples thickness variation before and after soaking processes, as well as after hydraulic conductivity tests, was recorded.

The hydraulic conductivity test for specimens soaked (soak time = 20 days) and permeated with water was conducted under the same conditions to serve as the contrast of the tests using solutions. These test results for water were compared with those of the solutions to obtain the different impacts of water and solutions on the variation law of GCL hydraulic conductivity. 

The hydraulic conductivity tests were operated in accordance with ASTM D5084 and ASTM D5887 [19,20].

## 3. Results and Discussion

### 3.1. GCL Hydraulic Conductivity and Thickness Variation Results under Water Soaking and Permeation

**Figure 1:**
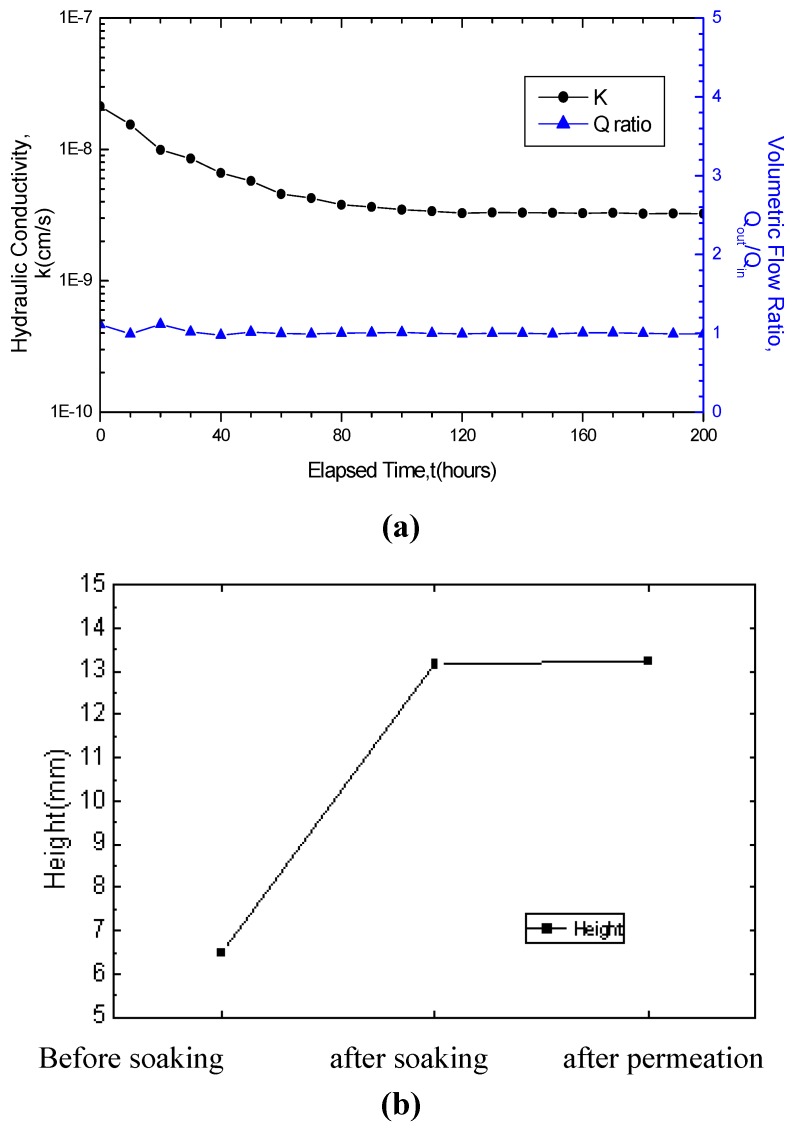
Test results for GCL specimens permeated with water: (**a**) variation in hydraulic conductivity; (**b**) variation in thickness.

Figure 1a and b present the curve of the GCL hydraulic conductivity variation with the time and the curve of the GCL thickness variation before after soaking and after permeation using water respectively. As seen in Figure 1a the GCL hydraulic conductivity fluctuated in the first five days, decreased from 2.13 × 10^−8^ cm/s to 3.25 × 10^−9^ cm/s and then became stable. The large decrease of GCL hydraulic conductivity was due mainly to the extremely significant hydration swelling of bentonite. This indicates that the hydration swelling of GCL still progressed in the initial period and finished after five days. The fluctuation of the Q_out_/Q_in _ratio curve at the first several times also confirmed this conclusion. In Figure 1b, the GCL thickness increased after soaking from 6.486 mm to 13.16 mm, indicating an evident expansion effect. By hydration swelling effect, the bentonite in the GCL formed colloids with high cohesion and became denser because of the restriction of the geotextiles and needle-punched fiber. Several colloids entered into the bentonite pore under inflation pressure, thereby blocking the water channel in the liner and further enhancing its impermeability. 

### 3.2. GCL Hydraulic Conductivity and Thickness Variation Results under Solution Soaking and Permeation

#### 3.2.1. Impact of Solution Categories on GCL Hydraulic Conductivity

**Figure 2:**
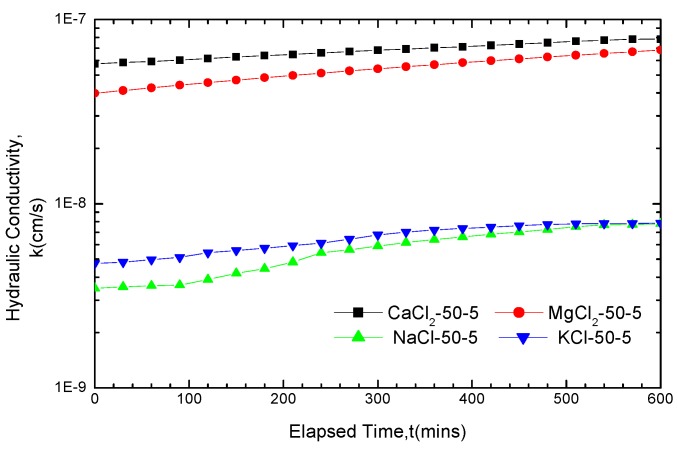
Hydraulic conductivity results for GCL specimens soaked with 50 mM solutions for five days and permeated with the same solutions after soaking.

**Figure 3:**
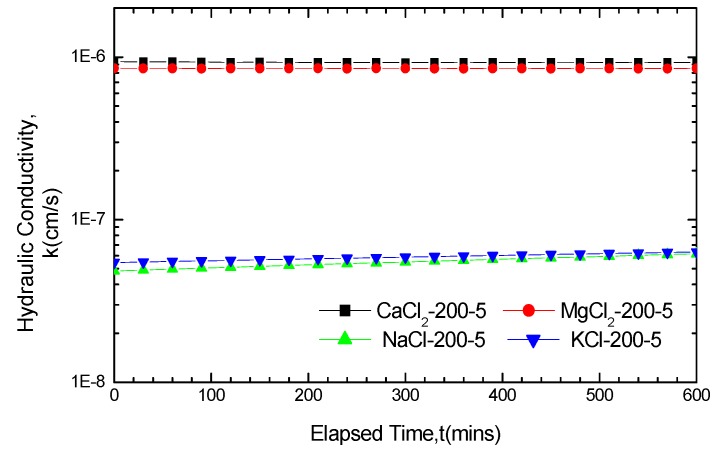
Hydraulic conductivity results for GCL specimens soaked with 200 mM solutions for five days and permeated with the same solutions after soaking.

The curves of the hydraulic conductivity variation for GCL soaked for five days in the four solutions with concentrations of 50 mM and 200 mM are presented in Figure 2 and Figure 3. 

By comparing Figure 2 and 3 with Figure 1a the hydraulic conductivity of the GCLs soaked in solutions for permeation were found to achieve different degrees of increase than that soaked in water. The increase in amplitude varies from several times to exceeding two orders of magnitude. If soaked in the 200 mM solutions for five days, the final hydraulic conductivity of GCLs permeated with CaCl_2_, MgCl_2_, KCl and NaCl solutions are 9.25 × 10^−7^ cm/s, 9.25 × 10^−7^ cm/s, 6.33 × 10^−8^ cm/s and 6.17 × 10^−8^ cm/s, respectively, which increased 284 times, 262 times, 19.5 times and 19.0 times, respectively, compared with the hydraulic conductivity, 3.25 × 10^−9^ cm/s, under water conditions. This finding revealed the evident chemical attack effect on the GCLs of high concentration solutions.

The curve of the hydraulic conductivity variation indicated large fluctuations, demonstrating a certain increase between the original and final points. Figure 2 presents the curve of hydraulic conductivity variation. From the original point to final point, the hydraulic conductivity under CaCl_2_, MgCl_2_, KCl, and NaCl solutions increased from 5.75 × 10^−8^ cm/s to 7.84 × 10^−8^ cm/s, from 3.98 × 10^−8^ cm/s to 6.84 × 10^−8^ cm/s, from 4.74 × 10^−9^ cm/s to 7.86 × 10^−9^ cm/s and from 3.48 × 10^−9^ cm/s to 7.79 × 10^−9^ cm/s, respectively, achieving an increase of 2.09 × 10^−8^ cm/s, 2.86 × 10^−8^ cm/s, 3.12 × 10^−9^ cm/s and 4.31 × 10^−9^ cm/s, respectively. The increase equaled 36.3%, 71.8%, 65.8% and 123.8% of the solutions hydraulic conductivity at the original points, respectively. In view of the increased percentages, the hydraulic conductivity under MgCl_2_ and NaCl achieved greater increase, whereas the increase under CaCl_2_ and KCl was smaller. The curve of hydraulic conductivity variation in Figure 3 produced only small fluctuations and even remained stable. Nevertheless, the increased percentages of the hydraulic conductivity under KCl and NaCl were larger than that of CaCl_2_ and MgCl_2_.

The GCL hydraulic conductivity results under different environmental conditions at the end of the tests were shown in Table 3. Based on the comparison of hydraulic conductivity under different solutions in Table 3, the GCL chemical attack effect was closely related with solution categories. With the same solution concentrations and soak times, the order of chemical attack degree was CaCl_2_ > MgCl_2_ > KCl > NaCl.

**Table 3:** Hydraulic conductivity of GCL specimens in each working condition.

Hydraulic conductivity k/10^−7cm/s^		200-5	200-10	200-20	100-5	100-10	100-20	50-5	50-10	50-20
solutions	CaCl_2_	9.25	8.99	8.96	6.85	7.73	6.82	0.784	0.912	1.06
	MgCl_2_	8.52	8.17	7.84	4.57	5.65	6.31	0.684	0.771	0.835
	NaCl	0.617	0.643	0.532	0.0988	0.217	0.272	0.0779	0.115	0.171
	KCl	0.633	0.715	0.656	0.124	0.287	0.323	0.0786	0.147	0.205

#### 3.2.2. Impact of Solution Concentrations on GCL Hydraulic Conductivity

**Figure 4:**
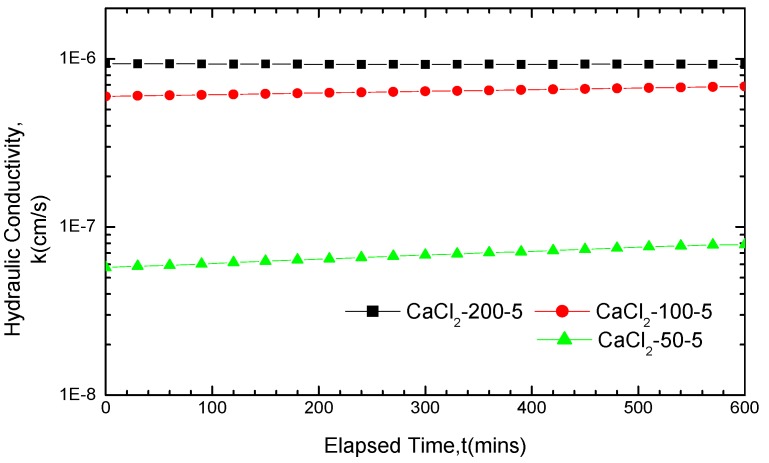
Hydraulic conductivity results for GCL specimens soaked with CaCl_2_ solution for five days and permeated with the same solution after soaking.

**Figure 5:**
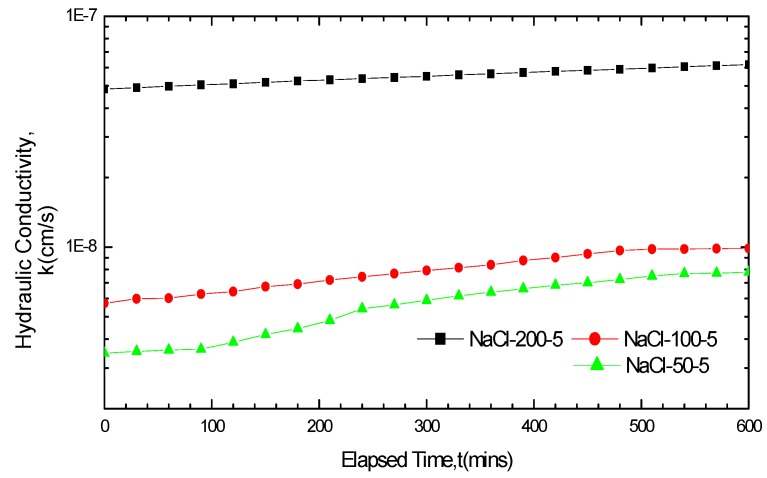
Hydraulic conductivity results for GCL specimens soaked with NaCl solution for five days and permeated with the same solution after soaking.

The variations of the GCL hydraulic conductivity with time after soaking in CaCl_2_ and NaCl solutions for five days are plotted in Figure 4 and Figure 5, respectively. These curves indicated that the GCL hydraulic conductivity was positively correlated with the solution concentration when soaked in the same solution for five days. Viewed from the data in Table 3, the GCL hydraulic conductivity, corresponding to three concentrations, revealed a great difference under the condition of the same solution and soak time. The maximum difference even exceeded one order of magnitude.

The GCL hydraulic conductivity basically remained stable from the beginning of permeation when soaked in the 200 mM CaCl_2_ solution, as seen in Figure 4. The hydraulic conductivity variation curve can be viewed as a straight line parallel to the abscissa axis. However, the GCL hydraulic conductivity increased gradually over time under the 50 mM and 100 mM concentrations. The curve of the GCL hydraulic conductivity variation achieved some increase under the concentration of 200 mM based on the NaCl solution in Figure 5. The stability degree of the hydraulic conductivity variation curve in Figure 4 and Figure 5 followed the order 200 mM > 100 mM > 50 mM. This finding indicated that a higher solution concentration resulted in a smaller variation in the GCL hydraulic conductivity with time, which meant a shorter time for the hydraulic conductivity variation curve to get stable and gentler. The increase or fluctuation of the GCL hydraulic conductivity was mainly caused by the incomplete equilibrium of the chemical attack reaction between the solutions and the liners under these conditions. The time it took to achieve equilibrium depended significantly on the concentration of solutions.

#### 3.2.3. Impact of Soak Times on GCL Hydraulic Conductivity 

According to the data in Table 3, the GCL hydraulic conductivity decreased to 8.96 × 10^−7^ cm/s, from 9.25 × 10^−7^ cm/s and 8.99 × 10^−7^ cm/s when soaked in the 200 mM CaCl_2_ solution for 20 days than for five days and ten days, respectively. The data for MgCl_2_ solution also demonstrated a decreasing trend. These results were mainly due to the uncompleted hydration swelling of the GCL when the chemical attack reaction had already got equilibrium. However, for the NaCl solution, the hydraulic conductivity increased from 6.17 × 10^−8^ cm/s to 6.43 × 10^−8^ cm/s, and decreased afterwards to 5.32 × 10^−8^ cm/s. And for the KCl solution, the same trend was achieved. Given the 200 mM concentration with the soak time extending from five days to ten days, the variation of the hydraulic conductivity was the result of the cooperation effect of the solution chemical attack reaction and the hydration swelling. During this period, the chemical attack reaction was relatively stronger; thus, increasing the hydraulic conductivity. However, the chemical attack reaction stabilized afterwards while the hydration swelling was still progressing. This phenomenon decreased the hydraulic conductivity from 200–10 to 200–20. When the solution concentration was 100 mM or 50 mM, the GCL hydraulic conductivity was positively correlated with the soak time because the solution chemical attack had not achieved equilibrium and was still reacting. The 100 mM CaCl_2_ solution which had the same variation law with the 200 mM NaCl and KCl solutions was not included.

**Figure 6:**
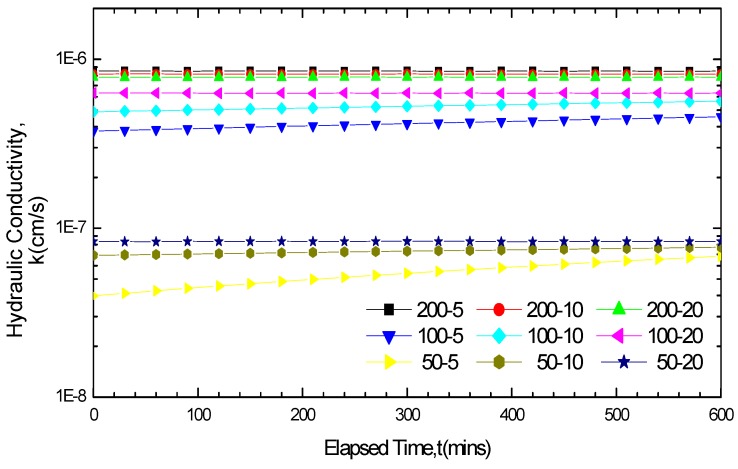
Hydraulic conductivity results for GCL specimens soaked and permeated with MgCl_2_ solution.

**Figure 7:**
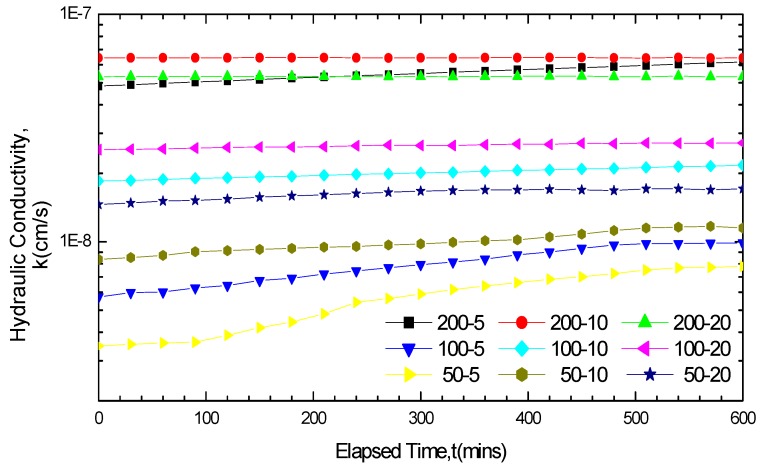
Hydraulic conductivity results for GCL specimens soaked and permeated with NaCl solution.

The hydraulic conductivity test results of MgCl_2_ and NaCl are shown in Figure 6 and Figure 7 respectively. The GCL hydraulic conductivity variation curves increased under short soak times and small solution concentrations, but remained stable without fluctuations under long soak times and large solution concentrations. When the GCLs were soaked in the solutions for five days, the hydraulic conductivity variation curves showed an upward trend with a relative large increase except for the 200 mM MgCl_2_ solution. If the soak time was prolonged to 20 days, only the hydraulic conductivity variation curves of 50 mM and 100 mM NaCl achieved small increased fluctuations. Concerning the soak time of 10 days, the variations in the hydraulic conductivity with time under different conditions were correlated with the solution categories and concentrations, the hydraulic conductivity in some conditions remained stable, whereas in other conditions achieved increases. Fundamentally, the differences of the GCL hydraulic conductivity variation under different soak times were related with the equilibrium of the solution chemical attack reaction. The variation curves remained stable only when the reaction got equilibrium after soaking.

#### 3.2.4. Variation Law of GCL Thickness before and after Soaking

The GCL thickness variations before and after soaking in various solutions are presented in Table 4. The variations revealed hydration swelling of the GCLs in a different extent after being soaked in four solutions. During the hydraulic conductivity tests, the GCL thickness remained basically stable, displaying only small differences after soaking. This was mainly because the solutions exerted weak chemical attack effect on the GCL after soaking. Meanwhile, the GCL produced no consolidation under the test conditions, thus leading to the unapparent thickness variation.

**Table 4:** Variations in thickness of GCL specimens after soaking.

variations in thickness Δ*h* /mm		200-5	200-10	200-20	100-5	100-10	100-20	50-5	50-10	50-20
solutions	CaCl_2_	0.11	0.21	0.215	0.645	0.53	0.685	2.28	2.075	2.015
	MgCl_2_	0.38	0.41	0.49	1.36	1.02	1.01	2.589	2.501	2.43
	NaCl	2.105	1.948	2.162	3.126	2.651	2.47	4.33	3.96	3.52
	KCl	1.824	1.615	1.79	2.847	2.165	2.03	4.095	3.675	3.16

The increasing amplitude of thickness revealed to be opposite of the hydraulic conductivity given different chemical solutions with the same concentrations and soak times. In other words, the GCLs achieved the largest thickness variation after soaking in NaCl solutions, then followed by KCl, MgCl_2_, and CaCl_2_ solutions. The thickness variations soaked in chemical solutions were all smaller than that soaked in water. This finding indicated that chemical attack restrained the GCL hydration swelling in a certain extent and weakened its swelling. The weakening effect degree followed this order: CaCl_2_ > MgCl_2_ > KCl > NaCl. The concentration of chemical solutions was negatively correlated with the increasing amplitude of thickness. Thus, larger chemical solution concentrations resulted in less GCL hydration swelling.

**Figure 8:**
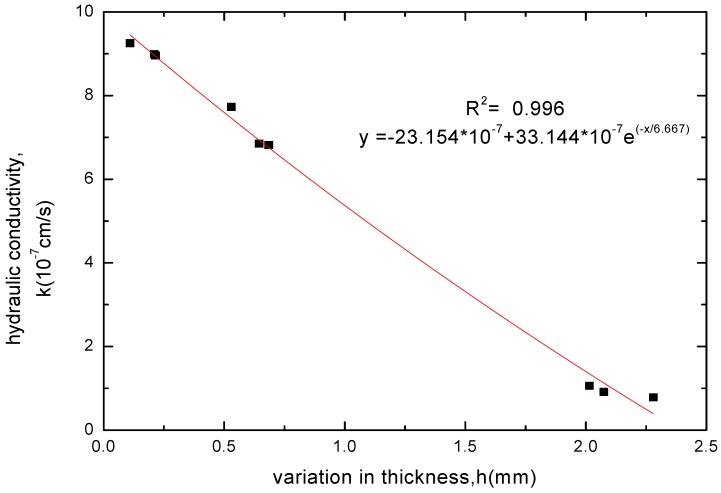
The relationship between hydraulic conductivity and thickness increase for GCL specimens soaked with CaCl_2_ solution.

**Figure 9:**
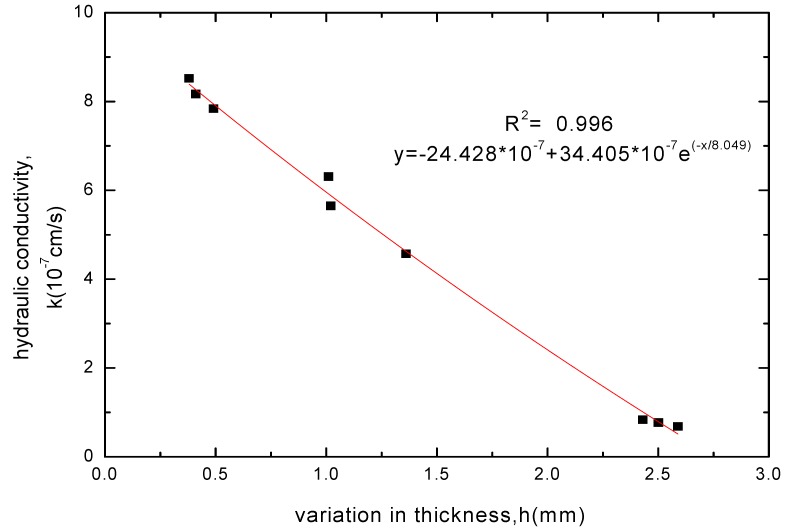
The relationship between hydraulic conductivity and thickness increase for GCL specimens soaked with MgCl_2_ solution.

**Figure 10:**
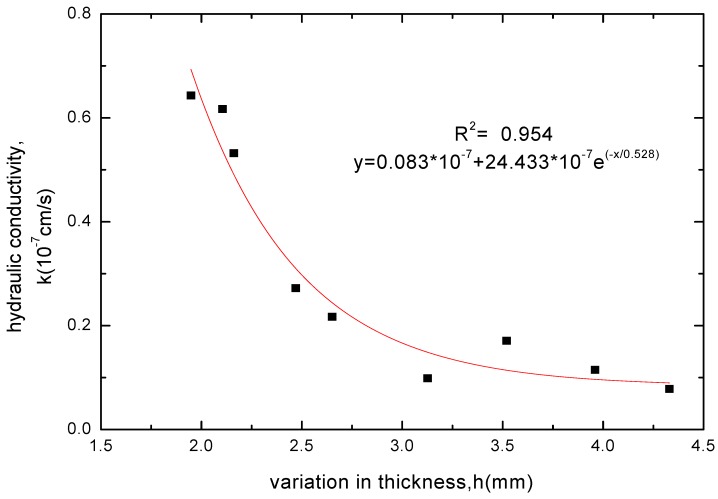
The relationship between hydraulic conductivity and thickness increase for GCL specimens soaked with NaCl solution.

**Figure 11:**
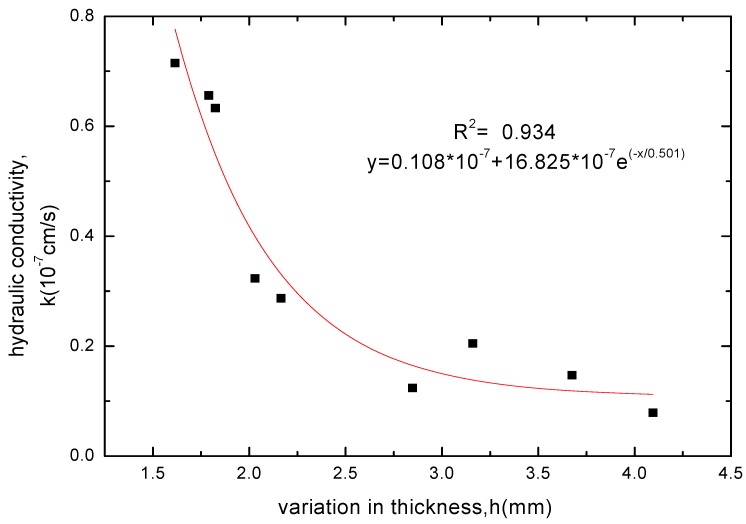
The relationship between hydraulic conductivity and thickness increase for GCL specimens soaked with KCl solution.

The GCL thickness increase after soaking was related to the variation of soak times and whether the chemical attack reaction of solutions had got equilibrium. When the chemical attack reaction of solutions had not got equilibrium and played the superior role, the GCL thickness decreased with extending soak time. However, the GCL thickness achieved a slight increase through hydration swelling when soaked continuously after the chemical attack reaction got equilibrium. Therefore, the GCL thickness increase after soaking was corresponded to the hydraulic conductivity. The relationships between the GCL thickness increase under four chemical solutions and the hydraulic conductivity were shown in Figure 8, Figure 9, Figure 10 and Figure 11. The tests results indicated that the hydraulic conductivity exponentially decreased with the increased thickness showing as follows:

(1) $${\text{CaCl}}_{\text{2}}\text{: }y=-23.154\times 10^{-7}+33.144\times 10^{-7}e^{-x/6.667}$$

(2) $${\text{MgCl}}_{\text{2}}\text{: }y=-24.428\times 10^{-7}+34.405\times 10^{-7}e^{-x/8.049}$$

(3) $$\text{NaCl: }y=0.083\times 10^{-7}+24.433\times 10^{-7}e^{-x/0.528}$$

(4) $$\text{KCl: }y=0.108\times 10^{-7}+16.825\times 10^{-7}e^{-x/0.501}$$

### 3.3. Analysis of pH and EC Test Results

**Figure 12:**
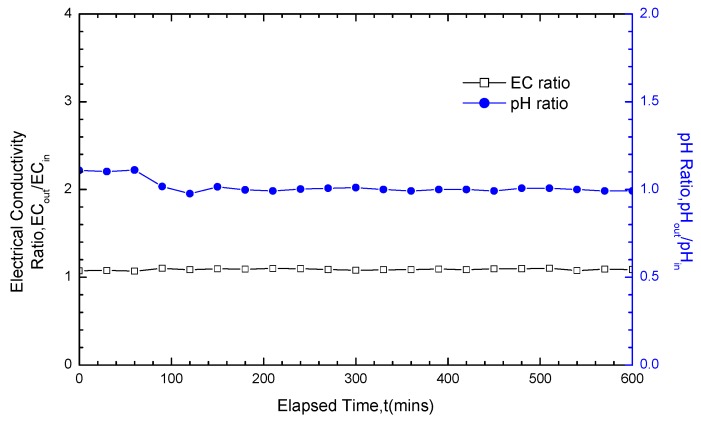
pH ratio and EC (electrical conductivity) ratio results for GCL specimens soaked with 200 mM CaCl_2_ solution for 20 days and permeated with the same solution after soaking.

**Figure 13:**
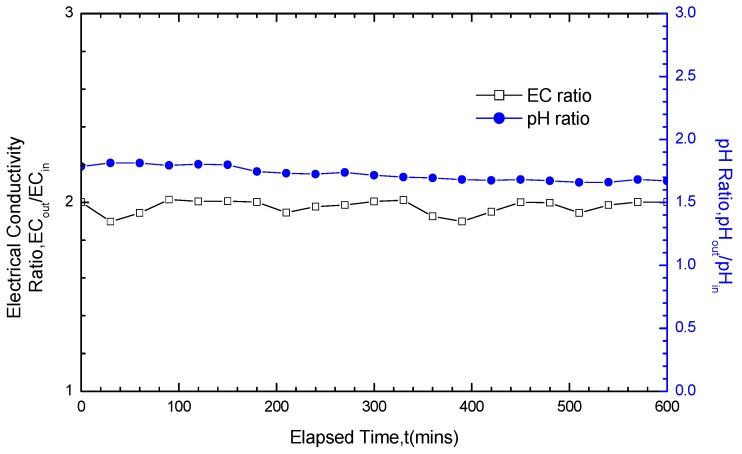
pH ratio and EC (electrical conductivity) ratio results for GCL specimens soaked with 50 mM NaCl solution for five days and permeated with the same solution after soaking.

Except for the consideration of whether fluctuations on the hydraulic conductivity variation curves existed, the test results also depended on the pH ratio as well as EC ratio between the outlet and inlet (pH_out_/pH_in_ and EC_out_/EC_in_) to determine whether the chemical attack reaction got equilibrium. Both pH_out_/pH_in_ and EC_out_/EC_in_ should be within a 1.0 ± 0.1 when equilibrium builds. Under large solution concentrations and long soak times (for example, the 200 mM CaCl_2_ solution with soak time of 20 days, as shown in Figure 12), both pH_out_/pH_in_ and EC_out_/EC_in_ were able to satisfy the equilibrium requirement. On the contrary, under small concentrations and short soak times, the GCLs did not achieve equilibrium after soaking and was under chemical attack before the termination of hydraulic conductivity tests (for example, the 50 mM NaCl solution with soak time of five days, as shown in Figure 13). This finding was not only in good accordance with the test results of the hydraulic conductivity variation curve fluctuation, but also fully confirmed the reliability of the test results on the variation law of GCL hydraulic conductivity under solutions soaking. 

### 3.4. Analysis and Discussion of the Test Results

The bentonite layer in GCL determines the hydraulic conductivity. The applied sodium-bentonite in GCL was mainly comprised of Na montmorillonite (76.3%). The increasing of GCL hydraulic conductivity after solution soaking and permeation was mainly related to the thickness of electric double layer on the montmorillonite mineral surface. According to the theory of electric double layer [21], the interaction between montmorillonite minerals particles and cations produces one layer of closely arranged cations and the other layer of diffused and distributed cations on the particles surface. These two layers produced from the interaction are known as the absorbed layer and diffused layer, respectively. A thicker electric double layer results in smaller GCL hydraulic conductivity and better impermeability. Its calculation formula is [22]:

(5) $$\lambda =\sqrt{\frac{\varepsilon \varepsilon_{0}RT}{2\nu^{2}F^{2}\eta }}$$

In Equation (5), *λ* is Debye length. *ε* is relative dielectric constant. *ε_0_* is vacuum dielectric constant, 8.854 × 10^−12^ *c*^*2*^/*J m*. *R* is Boltzmann constant, 8.314 *J/mol K*. *T* is thermodynamic temperature, K. *v* is cation valence. *F* is Faraday constant, 9.6487 × 10^4^*c/mol*, *η* is concentration of electrolyte, *mol/m^3^*.

Based on the combination of the test results and the aforementioned theoretical analysis, Na^+^ in the montmorillonite layer exchanges with bivalent ions (Ca^2+^ or Mg^2+^) in the solutions when CaCl_2_ or MgCl_2_ is applied for experiments. This cation-exchange reaction will increase the valence of cations on surface of montmorillonite mineral particles. In view of Equation (5), the thickness of the electric double layer is inversely proportional to the valence of cations as well as the square root of the electrolyte concentration. The thickness of the electric double layer decreased with the exchange of Ca^2+^ or Mg^2+^, thus widening the effective channel for liquid transmission in GCL and increasing its hydraulic conductivity. When soaked in NaCl and KCl solutions for permeation, monovalent cation (Na^+^ or K^+^) hardly produces cation exchanges. Therefore, the main reason for the GCL hydraulic conductivity increase lies in the solution concentration variations.

Except for the solution concentrations factor, the GCLs soaked in CaCl_2_ and MgCl_2_ solutions also suffered the impact of cation exchange reaction. So the CaCl_2_ and MgCl_2_ solutions exerted a stronger chemical attack effect on GCLs than NaCl and KCl solutions. The cooperation effect of the solution chemical attack reaction and the hydration swelling resulted in the variation of the GCL thickness. As the chemical attack reaction restrained the swelling of the GCL, continuous soaking weakened the chemical attack reaction and revealed the effect of hydration swelling. Therefore, whether the chemical attack reaction achieved equilibrium after soaking was the determinant of the GCL increased thickness varied with soak times. The increased thickness amplitude indicated that the chemical attack reaction achieved equilibrium while the hydration swelling was still in progress. In contrast to the cases with long soak times, the cases with short soak times revealed an opposite trend.

## 4. Conclusions

This paper presents the following main conclusions through experimental research on the GCL hydraulic properties under chemical attack by high concentration solutions:

(1) The GCL hydraulic conductivity is significantly influenced by chemical solutions with high concentrations. The GCL hydraulic conductivity under tests using chemical solutions achieves an increase from several times to over two orders of magnitude compared with that using water.

(2) For CaCl_2_, MgCl_2_, KCl and NaCl solutions, chemical solutions with high-valence cations exert a stronger chemical attack effect on GCL than those with low-valence cations. The CaCl_2_ solutions exert the strongest effect and then followed by MgCl_2_, KCl and NaCl.

(3) Given the same chemical solutions with fixed soak times, the GCL hydraulic conductivity corresponding to 50, 100 and 200 mM produce a significant difference. This reveals the positive correlation between hydraulic conductivity and the solution concentration. The increasing concentration thins the electric double layer on the GCL mineral particle surface and enlarges the hydraulic conductivity.

(4) The impact of soak times on GCL hydraulic conductivity is related to the cooperation effect of the solution chemical attack reaction and the hydration swelling. The chemical attack reaction does not achieve equilibrium under a short soak time. However, the hydration swelling becomes gradually advantageous as the soak time extends.

(5) Chemical solutions restrain GCL hydration swelling to a certain extent, and the GCL thickness increases after soaking in solutions far smaller than that in water. The hydraulic conductivity exponentially decreased with the increased thickness.
